# Population attributable risk for colorectal and breast cancer in England, Wales, Scotland, Northern Ireland, and the United Kingdom

**DOI:** 10.12688/amrcopenres.12980.2

**Published:** 2022-03-07

**Authors:** Shatabdi Goon, Hanseul Kim, Edward L. Giovannucci

**Affiliations:** 1 Department of Human Nutrition, University of Illinois at Urbana-Champaign, Urbana, IL, 61801, USA; 2 Florida Department of Health, Pasco, FL, 34652, USA; 3 Department of Epidemiology, Harvard T.H. Chan School of Public Health, Boston, MA, 02115, USA; 4 Department of Nutrition, Harvard T.H. Chan School of Public Health, Boston, MA, 02115, USA

**Keywords:** Population Attributable Risk, Colorectal Cancer, Breast Cancer, Risk Factors, Exposure Prevalence, Relative Risk, Cancer Prevention, United Kingdom.

## Abstract

**Background:**

The population attributable risk (PAR) is a statistic commonly used for quantifying preventability of cancer. We report here PAR estimates for the United Kingdom (UK) along with its constituent countries for up-to-date risk factor-attributable colorectal cancer (CRC) and breast cancer (BC), focusing on diet and nutrition related factors and tobacco (CRC) using representative national surveys.

**Methods:**

The PAR was calculated using established, modifiable risk factors by the World Cancer Research Fund/American Institute of Cancer Research (WCRF/AICR): physical activity, body mass index (BMI), alcoholic drinks, red meat, processed meat, dietary fiber, dietary calcium, as well as cigarette smoking for CRC, and physical activity, BMI, alcoholic drinks, and fruits and vegetable consumption for BC. National prevalence estimates and relative risks (RRs) for CRC and BC were obtained from meta-analyses or large pooled analyses.

**Results:**

Based on eight dietary and lifestyle risk factors, the estimates for attributable cases of CRC for males and females, respectively, were as follows: England: 67% and 60%; Scotland: 68% and 59%, Wales: 66% and 61%; Northern Ireland: 67% and 61%; and UK: 67% and 60%. Excluding smoking, the PAR for the UK was 61% for men and 52% for women. Based on four dietary and lifestyle risk factors, the estimates for BC were as follows: England: 26%, Scotland: 27%; Wales: 25%; Northern Ireland: 26%; and UK: 27%.

**Conclusion:**

Up to 67% for CRC and 27% of BC were attributable to modifiable dietary and lifestyle factors in the UK. Moderate differences in PAR are observed between countries due to different prevalence of exposure to risk factors.

## Plain language summary

A number of lifestyle factors, such as smoking tobacco, heavy alcohol, physical inactivity, excess body weight, and poor diet can cause cancer. Estimating the future preventable burden of cancers through modifications to these behaviors is critical. Population attributable risk has been extensively used in the literature for quantifying the preventable burden of cancer. Based on some dietary and lifestyle risk factors, we report here United Kingdom (UK) cancer preventability estimates along with its constituent countries on the number of risk factor-attributable colorectal cancer and breast cancer.

## Introduction

The population attributable risk (PAR) is a metric commonly utilized to quantify the preventability of a specific disease. Various approaches to calculate the PAR for cancer have been used by researchers. Most typically, the PAR is based on identifying relative risks (RRs) from the scientific literature and prevalence from suitable population sources for specific factors of interest. The process requires several steps. It is critical to first identify the established risk factors that are in principle modifiable. Then it is important to derive a RR estimate from the literature for each risk factor. The RR estimates can either be from the population of interest, or common RRs, such as from meta-analyses or representative studies, that are generalizable. Next, it is important to identify estimates of the prevalence of exposures in the population of interest. With this information, standard formulae to calculate PARs can be used.

Estimates of the PAR for various cancers have often varied widely. Four decades ago, Doll and Peto ^1^ suggested that 90% of colorectal cancers (CRCs) and 50% of breast cancers (BCs) may be related to diet. Similarly, Parkin *et al.* ^2^ attributed about 43% of CRC in the UK to five largely modifiable factors including diet and nutrition, and 42% of BC to modifiable risk factors including body fatness and physical activity. PAR estimates for cancer from studies across the world have varied substantially; for example, some estimates were between 16% and 90% for CRC ^3– 5^, and 6.5% and 50% for BC ^1– 3, 6^. Blot and Tarone ^7^ argued that an estimate of 90% appeared to be too high for CRC. Such variability in the calculated PAR estimates may extend from a number of factors ^8^ including: 1) the risk factors that were considered; 2) the specific RRs that were utilized; 3) the sources of population prevalence of the risk factors; and 4) the specific calculation methods used to calculate the PAR. Other contributors to variability include variation in the time period and geography, differences in socio-demographic profile of cancer cases, and differences due to screening availability.

We report here PAR estimates for the United Kingdom (UK) along with its constituent countries on the number of risk factor-attributable CRC and BC, the two most common preventable cancers, excluding lung cancer, which is highly related to tobacco use. We focused on diet- and nutrition-related factors (and tobacco for CRC) based on up-to-date criteria from the World Cancer Research Fund/American Institute of Cancer Research (WCRF/AICR) ^9^. We used representative national surveys for risk factor prevalence in individual UK countries given that the risk factor prevalence varies ^10^. Hence, we estimated the PARs for CRC and BC in the UK, and separately, England, Scotland, Wales, and Northern Ireland.

## Methods

### Factor selection

Based on criteria from the WCRF/AICR ^9^, we used only factors classified as having achieved a level of evidence for a causal association as “probable” or “convincing” (displayed in Table 1). This level of evidence is considered “actionable” by the WCRF/AICR. In addition to the seven factors that met this classification for CRC (body mass index (BMI), physical activity, alcoholic drinks, and intakes of dietary fiber, red meat, processed meat, and dietary calcium), we further included cigarette smoking, considered a causal factor for CRC based on the United States Surgeon General Report ^11^. We included four factors for BC (body fatness/BMI, physical activity, alcoholic drinks, and intakes of fruits and vegetables). We included “fruits and vegetables” because, although these were considered a limited/suggestive factor for BC, the WCRF/AICR expert panel concluded that that evidence for carotenoid rich foods was stronger for estrogen receptor negative BC than for estrogen receptor positive or total BC. Yet, because estrogen receptor negative BC is an important component of total BC, preventing it would contribute to reducing the burden of BC.

**Table 1.** Risk factors associated with increased colorectal cancer and breast cancer incidence considered in this study.

Exposure	Exposure category **	Cancer site (ICD-10)
Colorectal cancer *		
Cigarette smoking	**Never** Former Current	Colorectum (C18-C20)
Body fatness/BMI	**Normal (18.5-< 25 kg/m ^2^)** Overweight (25-29.9 kg/m ^2^) Obese (≥ 30 kg/m ^2^)	Colorectum (C18-C20)
Alcoholic drink	**None** Light (0.1-12.5 g/day) Moderate (12.6-50 g/day) Heavy (> 50 g/day)	Colorectum (C18-C20)
Insufficient physical activity	Inactive, less than 5 days of at least 30 minutes activity per week or < 600 MET-m/week **Active, more than 5 days of at least 30 minutes of activity or ≥ 600** **MET-m/week**	Colon (C18)
Processed meat	< 25 g/day vs. ≥ 25 g/day	Colorectum (C18-C20)
*Red meat*	< 70g/day vs. ≥ 70 g/day	Colorectum (C18-C20)
*Insufficient dietary fiber*	per 1 gm deficit per day **≥ 30 g/day**	*Colorectum (C18-C20)*
*Low dietary calcium*	0-524 mg/day 525-699 mg/day 700-899 mg/day 900-1000 mg/day **≥ 1000 mg/day**	*Colorectum (C18-C20)*
Breast cancer *		
Body fatness/BMI	**Normal (18.5-< 25 kg/m ^2^)** Overweight (25-29.9 kg/m ^2^) Obese (≥ 30 kg/m ^2^)	Breast (C50)
Alcoholic drink	**None** Light (0.1-12.5 g/day) Moderate (12.6-50 g/day) Heavy (> 50 g/day)	Breast (C50)
*Insufficient physical activity*	Inactive, less than 5 days of at least 30 minutes activity per week or < 600 MET-m/week **Active, more than 5 days of at least 30 minutes of activity or ≥ 600** ** MET-m/week**	Breast (C50)
Insufficient fruits and vegetables	< 5 servings **≥ 5 servings or ≥400 g/day**	Breast (C50)

*For colorectal cancer, exposure selected based on summary of strong evidence on diet, nutrition, physical activity and the prevention of cancer 2018 by WCRF/AICR (probable and convincing; Italicized factors are probable factors) plus cigarette smoking; Whole grain (and colorectal cancer) excluded because it can be attributed to fiber; Dairy (and colorectal cancer) excluded because it can be attributed to calcium. For breast cancer, exposure selected based on summary of strong evidence on diet, nutrition, physical activity, and the prevention of breast cancer 2017 by WCRF/AICR (probable and convincing; Italicized factor is probable, underlined factor is limited/suggestive). Dietary fiber (and breast cancer) excluded because it can be attributed to fruits and vegetables combined. **Theoretical minimum in bold

### Prevalence data

The prevalence of exposures to risk factors in the populations was obtained from the nationally representative population surveys: the Health Survey of England, Scotland, Wales, and Northern Ireland ^12^. Exposure distribution data on dietary calcium and meat consumption were obtained from EPIC-Oxford cohort ^13^ and UK biobank cohort ^14^, respectively. The methods detailing the derivation of prevalence for most exposures have been described in Brown *et al.* ^12^.

### Relative risks

We identified RRs by conducting systematic searches in PubMed. Meta-analyses of cohort studies were the preferred source of RRs, then followed by pooled analyses of cohort studies and individual cohort studies. In some meta- and pooled analyses multiple estimates were reported, and sometimes more than one meta- or pooled analysis was available; in these circumstances, we selected the RRs based on characteristics most relevant to our study. In addition, selected RRs had to provide cut-points for the categories comparable to the exposure data available for UK and its constituents countries. The search string for the risk factors were: Tobacco (tobacco OR cigarette OR smoking OR environmental tobacco smoke OR second-hand smoke), Overweight and obesity (weight OR BMI OR body mass index OR obesity OR obese OR overweight OR adiposity OR body size), Alcohol (alcohol OR alcoholic OR ethanol), Fiber (fibre OR fiber), Processed and red meat (Meat OR bacon OR ham OR sausages OR jerky OR salami OR cured OR salted OR Red), Insufficient physical activity (physical OR activity OR exercise OR physically active OR sedentary), Calcium (Calcium OR Dairy), and Fruits and vegetables (Fruits OR Vegetables OR Fruits and Vegetables).

As described previously for CRC ^8^, we used the formula, $\exp(\frac{ln(X)}{A}*\text{B})$ , to calculate RRs of different units for dietary calcium and physical activity. For calcium and CRC, we calculated RRs for each of the following categories: 0–199 mg/day, 200–399 mg/day, 400–599 mg/day, 600–799 mg/day, 800–999 mg/day, and ≥ 1000 mg/day ^8^. To match the categories available for the UK prevalence data (Oxford-UK Cohort), we then computed average RRs for overlapping categories to match with the exposure prevalence data available on resulting the RRs for categories: 0–524 mg/day, 525–699 mg/day, 700–899 mg/day, 900–1000 mg/day, and >1000 mg/day (e.g., calculating an average RR for 0–199 mg/day, 200–399 mg/day, and 400–599 mg/day to match 0–524 mg/day). Similarly, for physical activity and CRC, we first calculated RRs for each of the categories: 0–249 MET-m/week, 250–499 MET-m/week, 500–749 MET-m/week, 750–999 MET-m/week, and ≥1000 MET-m/week ^8^. We then computed an average RR for the first two categories representing: achieving less than 600 METs-m/week or active less than 5 days per week/ not achieving 30 minutes of physical activity on 5 days per week and active more than 5 days per week ^8^, that matches well with the exposure prevalence data on the UK national health surveys (prevalence of physical activity was provided as days per week on which at least 30 minutes of moderate physical activity was completed). For physical activity and CRC, we took a weighted average of colon and rectal cancers (70% colon cancer and 30% rectal cancer ^15^) to calculate the RR. Such assumptions made for RRs could have resulted in some small differences in the results.

For the factors associated with increased risk (BMI, alcohol, red meat, processed meat), we used the lowest category as the reference group. For the protective factors (physical activity, fiber, fruits and vegetable, and calcium), we chose the highest category as the reference group, and PAR was calculated using the reciprocal of the RR. For studies that provided a linear dose-response relationship, the RRs were first transformed into a log scale, divided by the value, then exponentiated.

### Statistical analysis

For each of the risk factors, we identified n levels of exposure categories. We then estimated PAR using the following equation:

$$\frac{\sum_{i=1}^{n}\;P_{i}*(RR_{i}-1)}{\sum_{i=1}^{n}\;P_{i}*(RR_{i}-1)+1}$$

where *P _i_* is the exposure prevalence at the exposure category i and *RR _i_* is the corresponding RR of CRC or BC at exposure category i. The details for the categorizations of exposures are presented in Table 1.

We then estimated the preventability of CRC or BC that was attributable to the combined dietary and lifestyle risk factors the following equation:

$$\text{PAR}=1-\prod_{i=1}^{n}\left(1-PAR_{i}\right)$$

where *i* signifies the level of individual risk factors (i = 1,…, n).

For fiber intake, the PAR was directly obtained from Brown *et al.* because our estimate was based on the same prevalence data and RR ^12^. The PAR estimates were directly computed manually using the formulae.

### Sensitivity analysis

We conducted an additional analysis where we exclude probable factors (defined by WCRF/AICR) and kept only the convincing factors from the calculation of the PAR. For CRC, the three probable factors (red meat, dietary fiber, and dietary calcium) were excluded from the analysis. We calculated the proportion of BC attributable to lifestyle factors excluding suggestive (fruits and vegetable consumption) and probable factors (physical activity), resulting in these two factors: body fatness/BMI and alcoholic drinks.

## Results

The proportion of CRC cases attributable to lifestyle risk factors for the UK constituents’ countries were estimated as follows: 67% for British males and 60% for British females, 68% for Scottish males and 59% for Scottish females, 66% for Welsh males and 61% for Welsh females, 67% for Northern Irish males and 61% for Northern Irish females, and 67% for UK males and 60% for UK females, overall ( Table 2). The proportion of CRC cases attributable to lifestyle risk factors excluding cigarette smoking were 62% for British males and 53% for British females, 62% for Scottish males and 51% for Scottish females, 61% for Welsh males and 52% for Welsh females, 62% for Northern Irish males and 52% for Northern Irish females, and 61% for UK males and 52% for UK females ( Table 2). The intake of dietary fiber was the major contributor to the attributable CRC cases, accounting for 25% for males and 32% for females in the UK, followed by processed meat intake (14% for men and 10% for women). For alcoholic drinks, the PAR values were substantially higher for men than for women. 

**Table 2.** Preventability estimates for colorectal cancer and breast cancer in England, Scotland, Wales, Northern Ireland, and United Kingdom (UK).

Exposure	Population attributable risk, %									
	England		Scotland		Wales		Northern Ireland		UK	
	Men	Women	Men	Women	Men	Women	Men	Women	Men	Women
Colorectal cancer *										
Cigarette smoking	14.1	15.5	14.2	15.7	14.4	18.6	13.3	15.0	14.0	16.2
Physical activity	7.0	4.8	7.5	5.6	6.4	4.2	6.0	5.1	6.7	5.1
Body fatness/BMI	13.5	6.0	13.3	5.7	12.3	5.8	13.9	5.7	13.3	5.6
Alcoholic drinks	13.6	2.2	13.3	1.9	13.4	2.0	13.5	1.9	13.0	1.9
Processed meat	14.5	10.0	14.5	8.5	14.5	7.4	14.5	9.6	14.5	8.5
Red meat	9.0	7.4	9.0	7.4	9.0	7.4	9.0	7.4	9.0	7.4
Dietary fiber	24.6	31.6	26.8	33.3	25.2	32.2	26.9	33.3	24.9	31.8
Dietary calcium	6.7	7.4	6.7	7.4	6.7	7.4	6.7	7.4	6.7	7.4
**Total (w/o smoking)**	**61.6**	**53.3**	**62.4**	**51.4**	**60.8**	**52.0**	**62.1**	**52.4**	**61.2**	**52.4**
**Total estimate**	**67.0**	**60.0**	**67.8**	**59.0**	**66.3**	**61.1**	**67.4**	**61.0**	**66.6**	**60.0**
Breast cancer **										
Body fatness/BMI	NA	7.1	NA	6.7	NA	6.1	NA	6.6	NA	7.6
Alcoholic drink	NA	8.5	NA	7.8	NA	8.3	NA	8.2	NA	8.2
Physical activity	NA	7.3	NA	8.2	NA	6.3	NA	7.5	NA	7.5
Fruits and vegetables	NA	6.4	NA	7.2	NA	7.0	NA	6.8	NA	6.8
**Total estimate**	**NA**	**26.2**	**NA**	**26.7**	**NA**	**25.0**	**NA**	**26.1**	**NA**	**26.9**

*Exposure categories: Physical activity (less than 5 days of at least 30 minutes activity per week or <600_METs-m/week vs. more than 5 days of at least 30 minutes activity per week or ≥ 600 MET-m/week); Body fatness/BMI (Normal (18.5-< 25 kg/m ^2^), Overweight (25-29.9 kg/m ^2^), Obese (≥ 30 kg/m ^2^)); Alcoholic drink (None, light (0.1-12.5 g/day), moderate (12.6-50 g/day), heavy (> 50 g/day)); Red meat (<70 g/day vs. ≥ 70 g/day); Processed meat (<25 g/day vs. ≥ 25 g/day); Dietary fiber (per 1 gm deficit per day, ≥ 30 g/day); Dietary calcium (<700 mg/day vs. ≥ 700 mg/day); Cigarette smoking (Never, Former, Current) **Exposure categories: Physical activity (less than 5 days of at least 30 minutes activity per week or <600_MET-m/week vs. more than 5 days of at least 30 minutes activity per week or ≥ 600 MET-m/week); Body fatness/BMI (Normal (18.5-< 25 kg/m ^2^), Overweight (25-29.9 kg/m ^2^), Obese (≥ 30 kg/m ^2^)); Alcoholic drink (None, light (0.1-12.5 g/day), moderate (12.6-50 g/day), heavy (> 50 g/day)); fruits and vegetables (<5 servings/day vs. ≥5 servings or 400g/day)

The proportions of BC cases attributable to lifestyle risk factors were 26% for British women, 27% for Scottish, 25% for Welsh, 26% for Northern Irish, and 27% for UK women, overall ( Table 2). Alcohol was the largest contributor to the estimated attributable BC cases, accounting for 8% for females in UK, followed by the body fatness (7.6%) and insufficient physical activity (7%).

The estimates for the prevalence and the RRs for the various exposures used in our calculation are presented in Table 3 and Table 4.

**Table 3.** Distribution of colorectal cancer and breast cancer exposures in the UK by country and sex *.

Exposure	Exposure category/unit	Prevalence, %									
		England		Scotland		Wales		Northern Ireland		UK	
		Men	Women	Men	Women	Men	Women	Men	Women	Men	Women
BMI	Normal (18.5-< 25 kg/m ^2^) Overweight (25-29.9 kg/m ^2^) Obese (≥ 30 kg/m ^2^)	31 43 22	40 33 24	37 41 22	35 31 23	35 42 18	41 32 18	38 39 25	38 30 23	36.9 41.3 21.8	46.5 31.5 22
Cigarette smoking	Never Former Current	4 28 27	56 20 24	45 27 28	55 21 24	45 26 29	46 24 30	50 23 27	62 13 25	46 26 27.8	54.7 19.5 25.8
Alcohol	None Light (0.1-12.5 g/day) Moderate (12.6-50 g/day) Heavy (> 50 g/day)	16 44 34 12	20 54 26 2	27 42 35 11	41 58 24 1	12 45 38 10	20 57 24 2	24 44 36 11	30 56 25 2	9.4 43.8 35.8 11	17.4 56 24.8 1.8
Physical activity	% not achieving 150+ minutes moderate physical activity per week or active 5 days/week % achieving 150+ minutes moderate physical activity per week or active 5 days/ week	39 61	27 73	43 57	31 69	36 64	23 77	33 67	28 72	38 62	28 73
Dietary fiber	g per day	20	16	19	15	19	16	18	15	19	15.5
Processed meat	% eating processed meat ≥25 g per day	74	50	74	50	74	50	74	50	74	50
Red meat	% eating red meat ≥70 g per day	55	47	55	47	55	47	55	47	55	47
Dietary calcium	0-524 mg/day 525-699 mg/day 700-899 mg/day 900-1000 mg/day ≥ 1000 mg/day	5.9 11.5 19.1 11.0 52.4	6.4 12.0 22.3 11.6 47.8	5.9 11.5 19.1 11.0 52.4	6.4 12.0 22.3 11.6 47.8	5.9 11.5 19.1 11.0 52.4	6.4 12.0 22.3 11.6 47.8	5.9 11.5 19.1 11.0 52.4	6.4 12.0 22.3 11.6 47.8	5.9 11.5 19.1 11.0 52.4	6.4 12.0 22.3 11.6 47.8
Fruits and vegetables (g/day)	% not eating five or more portions or 400g of fruit and vegetables per day	72	68	83	78	78	75	85	73	80	73

*These estimates were prevalence calculated by Brown *et.al.* 2015 ^12^. Exposure distribution data was obtained from the Health survey of England, Scotland, Wales, and Northern Ireland, and National Diet and Nutrition Survey ^16^ except for dietary calcium and meat. Exposure distribution data on dietary calcium was obtained from the EPIC-oxford cohort ^13^. Exposure distribution data on meat consumption was obtained from the UK biobank cohort ^14^.

**Table 4.** Relative risks for the associations between risk factors and colorectal cancer and breast cancer.

	In original reports				In this analysis when our exposure categories were different from those in original reports		
Colorectal Cancer							
Exposure	Exposure category/unit	Region; Study design	Relative risk (95% CI)		Exposure category/unit	Relative risk (95% CI)	
			Men	Women		Men	Women
Cigarette smoking ^17^	Never Former Current	Worldwide, Meta-analysis	Reference 1.20 (1.10, 1.30) 1.40 (1.20, 1.70)	Reference 1.20 (1.10, 1.30) 1.60 (1.40, 1.90)	same	same	same
BMI ^18^	Normal (18.5-< 25 kg/m ^2^) Overweight (25-29.9 kg/m ^2^) Obese (≥ 30 kg/m ^2^)	Worldwide, Meta-analysis	Reference 1.17 (1.12, 1.22) 1.38 (1.32, 1.44)	Reference 1.07 (1.01, 1.14) 1.17 (1.06, 1.30)	same	same	same
Alcohol ^19^	None Light (0.1-12.5 g/day) Moderate (12.6-50 g/day) Heavy (> 50 g/day)	Worldwide, Meta-analysis	Reference 1.05 (0.95, 1.16) 1.21 (1.11, 1.32) 1.53 (1.30, 1.80)	Reference 1.00 (0.89, 1.01) 1.07 (0.99, 1.16) 1.24 (0.68, 2.25)	same	same	same
Physical activity ^20^	Higher vs. lower (90 ^th^ percentile vs. 10 ^th^ percentile)	Worldwide, Meta-analysis	0.84 (0.77, 0.91)	0.84 (0.77, 0.91)	Inactive, less than 5 days of at least 30 minutes activity per week or < 600 MET- m/week Active, more than 5 days of at least 30 minutes of activity or ≥ 600 MET-m/week	1.19 Reference	1.19 Reference
Dietary fiber ^3^	Per 1 gm deficit per day	Worldwide, Meta-analysis	1.03 (1.01,1.06)	1.03 (1.01,1.06)	same	same	same
Processed meat ^21^	Per 50 g per day/increase	Worldwide, report	Colon: 1.23 (1.11, 1.35) Rectum: 1.08 (1.00, 1.18)	Colon: 1.23 (1.11, 1.35) Rectum: 1.08 (1.00, 1.18)	< 25 g/day ≥ 25 g/day	Reference 1.23	Reference 1.23
Red meat ^21^	Per 100 g per day/increase	Worldwide, report	Colon: 1.22 (1.06, 1.39) Rectum: 1.13 (0.96, 1.34)	Colon: 1.22 (1.06, 1.39) Rectum: 1.13 (0.96, 1.34)	< 70 g/day ≥ 70 g/day	References 1.18	References 1.18
Dietary calcium ^22^	Per 200 mg/day increase	Worldwide, Meta-analysis	0.93 (0.88, 0.99)	0.93 (0.91, 0.95)	0-524 mg/day 525-699 mg/day 700-899 mg/day 900-999 mg/day ≥ 1000 mg/day	1.34 1.20 1.12 1.04 Reference	1.33 1.20 1.12 1.06 Reference
**Breast cancer**							
BMI ^23^ Females	Normal (18.5-< 25 kg/m ^2^) Overweight (25-29.9 kg/m ^2^) Obese (≥ 30 kg/m ^2^)	Worldwide, Meta-analysis	NA	Reference 1.10 (1.06, 1.13) 1.18 (1.12, 1.25)	same	same	same
Alcohol ^19^ Females	None Light (0.1-12.5 g/day) Moderate (12.6-50 g/day) Heavy (> 50 g/day)	Worldwide, Meta-analysis	NA	Reference 1.04 (0.99, 1.09) 1.23 (1.16, 1.32) 1.60 (1.23, 2.19)	same	same	same
Physical activity ^20^	Higher vs. lower (90th percentile vs. 10th percentile)	US and Europe; Pooled analysis	0.94 (0.90, 0.98)	0.94 (0.90, 0.98)	Inactive, less than 5 days of at least 30 minutes activity per week or < 600 MET- m/week Active, more than 5 days of at least 30 minutes of activity or ≥ 600 MET-m/week	1.29 Reference	1.29 Reference
Fruits and vegetables combined ^24^	Highest vs. Lowest; ≥ 5 servings or 400 g/d vs. 100 g/d (no studies with zero intake)	Worldwide, Meta-analysis	0.89 (0.80, 0.99)	0.89 (0.80, 0.99)	same	same	same

### Sensitivity analysis

The PARs for CRC based on the five “convincing” factors (body fatness/BMI, physical activity, alcohol, processed meat, and cigarette smoking) were 48% for UK males and 34% for UK females, and excluding smoking, these were 40% and 21%, respectively ( Table 5). After excluding the “probable” factors for BC, the PARs including the 2 factors (body fatness/BMI and alcoholic drinks) were 15% for UK females ( Table 5). 

**Table 5.** Additional analysis: Preventability estimates (PAR) for CRC and BC in the UK, excluding probable factors based on the WCRF/AICR.

Exposure *	Standard PAR, %									
*Colorectal Cancer*										
	England		Scotland		Wales		Northern Ireland		UK	
	Men	Women	Men	Women	Men	Women	Men	Women	Men	Women
**Cigarette smoking**	14.1	15.5	14.2	15.7	14.4	18.6	13.3	15.0	14.0	16.2
**Physical activity**	7.0	4.8	7.5	5.6	6.4	4.2	6.0	5.1	6.7	5.1
**Body fatness/BMI**	13.5	6.0	13.3	5.7	12.3	5.8	13.9	5.7	13.3	5.6
**Alcoholic drink**	13.6	2.2	13.3	1.9	13.4	2.0	13.5	1.9	13.0	1.9
**Processed meat**	14.5	10.0	14.5	10.0	14.5	10.0	14.5	10.0	14.5	10.0
**Total (w/o smoking)**	40.5	21.1	40.1	21.4	40.0	20.4	40.1	20.4	40.0	21.0
***Total estimate***	***49.0***	***33.4***	***48.5***	***33.7***	***47.8***	***35.0***	***48.1***	***32.4***	***48.3***	***33.7***
***Breast Cancer***										
**Body fatness/BMI**	7.1		6.7		6.1		6.6		7.6	
**Alcoholic drink**	8.5		7.8		8.3		8.2		8.2	
***Total estimate***	***14.9***		***13.9***		***13.9***		***14.3***		***15.2***	

*Exposure categories same as Table 1

## Discussion

We provided PAR estimates for the UK and its constituent country-level for diet and lifestyle risk factors where evidence for a causal role in CRC and BC development is probable/convincing based on WCRF/AICR systematic reviews. We estimated that 67% of CRC cases in men and 60% of cases for women in the UK, and 27% of BC cases were attributable to dietary and lifestyle risk factors assessed in adulthood. Excluding smoking from the calculation, these estimates for CRC were 61% for males and 52% for females. Significant differences in the CRC PAR by sex were observed for body fatness, alcohol, and fiber intake; these results were mostly driven by sex differences in the RR estimates, and partly by higher levels of alcohol drinking in men. Moderate differences in the PAR estimates were observed between countries due to different prevalence of exposure to risk factors. For instance, prevalence of obesity/body fatness was slightly lower in Wales compared to other countries in the UK, resulting in slightly lower PAR estimates of both CRC and BC for Wales overall. Similarly, the highest prevalence of not achieving 150 minutes of weekly moderate physical activity was observed among Scottish population, resulting in slightly higher PAR estimates of both CRC and BC in Scotland.

Previous studies have considered the PAR in the UK and its individual countries. The results we report here show overall consistency with those from similar studies, despite some differences in the risk factors considered, the time periods encompassed, and the RR sources. In one study to determine preventability in the UK (2018) adults aged 30 years and older, the PAR of CRC attributable to tobacco smoking, alcohol, intakes of meat and fiber, overweight and obesity, physical exercise, and ionizing radiation was 57% for men and 51% for women ^12^. In the same study, the PAR of BC attributable to alcohol drinking, intakes of fruits and vegetables, overweight and obesity was 27% in 2010 ^2^ and 23% in 2018 ^12^. These results were similar but slightly lower compared with our estimates. This other analysis ^12^ did not account for red meat or dietary calcium, considered as probable risk factors by WCRF/AICR, and included radiation and oral contraceptive use, which we did not consider as our analysis focused on the most up-to-date evidence from the WCRF/AICR for modifiable cancers under the domain of diet and nutrition.

Wide variations in PAR estimates were observed in some previous studies across the world. For example, in a study that examined overweight and obesity, physical inactivity, and low consumption of fruits and vegetables in relation to CRC risk worldwide, the estimated PAR was 13% ^25^. A report of alcohol, obesity and overweight, and physical inactivity as modifiable risk factors associated with CRC risk found that the PAR was 19% for the French population, and 21% for men and 16% for women ^5^. Although our estimates were for the UK rather than worldwide, they suggest that the PAR of 13% probably underestimates the true preventability because the overall CRC rates are not markedly different between France and the UK.

Our estimates of the PAR here were based on our judgement of the best available and most relevant data, and thus cautious interpretation is warranted. These limitations could bias either toward underestimation or overestimation of PARs. We have previously shown that the choice of risk factors and selection criteria for the sources of RRs could influence the PAR estimates, although in general, the methods appear relatively robust ^8^. We further performed sensitivity analysis by excluding probable factors to assess the influence of risk factor selection on PAR estimation. Yet this analysis is likely to considerably underestimate the true PAR because the strength of evidence for “probable” risk factors is high enough to be considered actionable by the WCRF/AICR. The associations classified as “probable” by the WCRF/AICR are robust, but, nevertheless, because they are based on observational studies, confounding cannot be discounted with certainty. Another limitation is, even if the considered factors are truly causal, it is unclear when in life they need to be altered to mitigate risk.

On the other hand, some considerations indicate that we may underestimate the true preventability. Most studies utilize a single measure based on a dietary or physical activity assessment with considerable measurement error; thus, any true associations would tend to be underestimated because random error typically causes a true association to tend towards the null value. Although BMI is a useful measure and generally measured well, it is not a perfect measure of the most relevant component of adiposity (e.g., visceral fat). The PARs for alcohol are prone to be underestimates because non-drinkers (the presumed low-risk group) may include past drinkers who may have consumed heavily before stopping to drink ^19^. Finally, the overall preventability over the life course is likely to be underestimated because we only considered adulthood diet. In fact, adolescence is emerging as an important time period of increased susceptibility to carcinogenic exposures, including diet, especially for BC but also for CRC.

In conclusion, our study reported the PAR of CRC and BC attributable to modifiable risk factors in England, Scotland, Wales, Northern Ireland, and UK. Up to 67% for CRC and 27% of BC were attributable to known established modifiable risk factors in adulthood in the UK. These results reinforce the importance for diet and lifestyle for the prevention of major cancers in the UK.

## Data availability

All data underlying the results are available as part of the article and no additional source data are required.
