# Supplementary material for: Population attributable risk for colorectal and breast cancer in England, Wales, Scotland, Northern Ireland, and the United Kingdom
Source: AMRC Open Res. 2022 Mar 7;3:11. Originally published 2021 Mar 23. [Version 2] doi: 10.12688/amrcopenres.12980.2 (PMC11064977; doi:10.12688/amrcopenres.12980.2)
Supplement: Peer review history

# Reviewer report: Reviewer response for version 2

**DOI:** 10.21956/amrcopenres.14143.r26951

**Published:** 2022-03-14

**Authors:** Yibing Ruan

**Affiliations:** 1 Department of Cancer Epidemiology and Prevention Research, Alberta Health Services, Calgary, AB, Canada

The authors have made some revisions and have largely acknowledged the potential biases of their PAR estimates and the limitations of their study. I have no further comments.

Is the work clearly and accurately presented and does it cite the current literature?

Partly

If applicable, is the statistical analysis and its interpretation appropriate?

Not applicable

Are all the source data underlying the results available to ensure full reproducibility?

Yes

Is the study design appropriate and is the work technically sound?

No

Are the conclusions drawn adequately supported by the results?

Partly

Are sufficient details of methods and analysis provided to allow replication by others?

Partly

Reviewer Expertise:

Cancer epidemiology; Population attributable risk; Biostatistics; Molecular epidemiology.

I confirm that I have read this submission and believe that I have an appropriate level of expertise to confirm that it is of an acceptable scientific standard.

# Reviewer report: Reviewer response for version 1

**DOI:** 10.21956/amrcopenres.14053.r26795

**Published:** 2021-09-24

**Authors:** María Teresa Álvarez Bañuelos

**Affiliations:** 1 Instituto de Salud Pública, Universidad Veracruzana, Veracruz, Mexico

This reports here the population attributable risk (PAR) estimates for the United Kingdom along with its constituent countries for up-to-date risk factor-attributable colorectal cancer (CRC) and breast cancer (BC), focusing on diet and nutrition related factors and tobacco (CRC) using representative national surveys.

 The manuscript is well-written and nicely presented, with a good balance of descriptive text and speech and practical tables of the use of the package.

 The methods used seem relatively robust but limited in their causality, they did not include incidence, although the study provides interesting data on the two important neoplasms in public health. However, it was limited to estimates of national prevalence only and the relative risks (RR) failed to include important socio-demographic risk factors such as age group for both CRC and CC.

 Discussion. The limitations of the study were mentioned, which are cautious in making strong statements regarding the results.   

 An important question would be about the validity of the study, the text does not say anything and about the robustness of the study, or if the research question was important and if the study it was original.

Is the work clearly and accurately presented and does it cite the current literature?

Yes

If applicable, is the statistical analysis and its interpretation appropriate?

Not applicable

Are all the source data underlying the results available to ensure full reproducibility?

Yes

Is the study design appropriate and is the work technically sound?

Partly

Are the conclusions drawn adequately supported by the results?

Yes

Are sufficient details of methods and analysis provided to allow replication by others?

Partly

Reviewer Expertise:

Epidemiology

I confirm that I have read this submission and believe that I have an appropriate level of expertise to confirm that it is of an acceptable scientific standard.

# Reviewer report: Reviewer response for version 1

**DOI:** 10.21956/amrcopenres.14053.r26735

**Published:** 2021-07-20

**Authors:** Yibing Ruan

**Affiliations:** 1 Department of Cancer Epidemiology and Prevention Research, Alberta Health Services, Calgary, AB, Canada

The authors provided some PAR estimates for the dietary and lifestyle risk factors associated with CRC and BC in UK along with its constituent countries. I couldn’t help wondering about the rationale underlying this study and several methodologic issues after reading this manuscript.

1. The rationale of carrying out this study eludes me, especially a large chunk of the result was just a repetition of Brown et al. (Br J Cancer, 2018, 118(8): 1130–41 ^1^). The PAR for individual and combined risk factors of CRC and BC can be found in the supplementary materials of Brown’s paper, except for dietary calcium and red meat. Was the rationale of this study to update the PAR estimates in UK by including these two risk factors, which were excluded in Brown’s paper?
2. The authors obtained exposure distribution data on dietary calcium and red meat consumption from EPIC-Oxford cohort and UK biobank cohort (page 3). However, these two cohorts are not population representative of UK. For example, the EPIC-Oxford cohort clearly stated on their website that the sample is not representative of the general population, as 50% of their sample are vegetarians. ( http://www.epic-oxford.org/faq/1262/frequently-asked-questions). Similarly, UK Biobank participants were more likely to be older, to be female, and to live in less socioeconomically deprived areas than nonparticipants (Fry A. et al., Am J Epidemiol. 2017, 186(9):1026-34 ^2^). Therefore, the PAR estimates of calcium and red meat are bound to be biased using the data.
3. I wonder why the authors did not obtain cancer incidence data from the cancer registries in UK. Without the incidence data, there are at least three limitations regarding the PAR estimates from this study. First, the authors could not estimate the attributable cancer cases, which is a constituent part of PAR estimation. Second, the authors could not do an age-stratified analysis. The summation of attributable cases of the age groups, divided by the cancer incidence of all age groups, provides a more accurate all-age PAR estimate than directly using an all-age prevalence to estimate PAR (the latter of which I suspect is what the authors did). This is because some risk factors are more prevalent among older age groups (e.g., physical inactivity, body fatness) and most cancer incidences are disproportionally higher among older age groups. Second, the authors had to assume a weighted average of 70% colon and 30% rectal cancers for physical activity and CRC (page 5), which wouldn’t be necessary if they had the incidence data by colon and rectum.
4. The authors didn’t correctly estimate the PAR of body fatness and physical activity for BC. These two risk factors are only associated with postmenopausal breast cancers, whereas the authors included premenopausal breast cancers as well.
5. The way the authors calculated RRs for calcium and physical activity is difficult to understand. They used a formula that assumes a loglinear dose-response relationship and calculates RR based on per unit RR, which is a usual practice in this field. Then for dietary calcium, they calculated an average RR for 0–199 mg/day, 200–399 mg/day, and 400–599 mg/day from their IJC paper (Kim H. et al., Int J Cancer, 2021, 148(12):2947-53 ^3^) to match 0–524 mg/day of the lowest category of exposure in this study (page 5, first paragraph). It was unclear to me how they averaged the RR and how they resolved the issue that the highest category (400-599mg/day) exceeds the upper limit (524 mg/day). Why not just take the midpoint value of this category (262 mg/day) and combine it with the risk per unit value? For example, for a reference calcium level of 1000 mg/day and per mg/day unit RR of r_mg, the RR for the category of 0-524 mg/day is r_mg^(1000-262).

Some other concerns:

1. The authors used a formula, exp(lnX/A *B) to calculate RR, while neither A nor B was defined. The authors referenced their recent publication in IJC for this formula (Kim H. et al., Int J Cancer, 2021, 148(12):2947-53 ^3^). But in their IJC paper, the formula was laid out as “RR_B = exp(log(RR_B)/A *B)”, which is simply wrong. “log(RR_B)” should be “log(RR_A)”. The authors should write an erratum to IJC to have it corrected.
2. In the second paragraph of the Method section, the author stated, “the prevalence of exposures to risk factors in the populations was obtained from the nationally representative population surveys” (page 3). However, which risk factors exactly were not stated. This caused some confusions to me, as I tried to figure out from which data source they obtained the prevalence of processed meat consumption.
3. In Table 2, there is no variation in PAR by countries for red meat and dietary calcium (page 6). It is unclear whether there is indeed no country-level variation for these two risk factors, or there were no prevalence data to support this analysis, or there was a data-entry mistake. The authors didn’t have any discussions around it. If there were no prevalence data to support this analysis, then the discussion around differences in PAR between countries should acknowledge this limitation.
4. At the end of the second paragraph of introduction (page 3), the authors stated that other contributors to the variability in calculated PAR estimates by different studies differences include “socio-demographic profile of cancer cases, and differences due to screening availability”. As the authors didn’t provide citations to support this statement, could the authors further elaborate on how these two factors contribute to the variability? In particular, it is difficult to fathom how screening availability, a factor related to the secondary intervention of CRC and BC, contributes to the PAR of diet and nutrition.
5. The authors used an equation that has been widely adopted for estimating combined risk factors (page 5, paragraph 4). However, many researchers used this formula without disclaiming the two important assumptions associated with it (Steenland and Armstrong, 2006, Epidemiology, 17(5):512-9 ^4^): independent risk exposure distributions and no statistical interactions between any two risks. These two assumptions should not only be explicitly stated in the method section, but also be worthy of several sentences in the discussion section. Also on page 5 after the formula, the author said “where i signifies the level of individual categories (i = 1,…, n).” “Categories” here should be “risk factors”. Again for this paragraph, the first sentence “We then estimated the preventability of CRC or BC that was attributable to the combined dietary and lifestyle risk factors the following equation” is missing a preposition.
6. Page 5 line 10, “600 METs-hours/week” should be “600 MET-m/week”.

In summary, I find little value being added by this paper beyond the study by Brown et al. (2018). The authors should justify the validity of their PAR estimates for dietary calcium and red meat and address all my concerns above before the paper could be considered having reasonable quality.

Is the work clearly and accurately presented and does it cite the current literature?

Partly

If applicable, is the statistical analysis and its interpretation appropriate?

Not applicable

Are all the source data underlying the results available to ensure full reproducibility?

Yes

Is the study design appropriate and is the work technically sound?

No

Are the conclusions drawn adequately supported by the results?

Partly

Are sufficient details of methods and analysis provided to allow replication by others?

Partly

Reviewer Expertise:

Cancer epidemiology; Population attributable risk; Biostatistics; Molecular epidemiology.

I confirm that I have read this submission and believe that I have an appropriate level of expertise to state that I do not consider it to be of an acceptable scientific standard, for reasons outlined above.

## References

[1] : The fraction of cancer attributable to modifiable risk factors in England, Wales, Scotland, Northern Ireland, and the United Kingdom in 2015. *Br J Cancer* .118(8) : 10.1038/s41416-018-0029-6 1130-1141 10.1038/s41416-018-0029-6 29567982 PMC5931106

[2] : Comparison of Sociodemographic and Health-Related Characteristics of UK Biobank Participants With Those of the General Population. *Am J Epidemiol* .2017;186(9) : 10.1093/aje/kwx246 1026-1034 10.1093/aje/kwx246 28641372 PMC5860371

[3] : A comparison of methods in estimating population attributable risk for colorectal cancer in the United States. *Int J Cancer* .2021;148(12) : 10.1002/ijc.33489 2947-2953 10.1002/ijc.33489 33527363 PMC8058283

[4] : An overview of methods for calculating the burden of disease due to specific risk factors. *Epidemiology* .2006;17(5) : 10.1097/01.ede.0000229155.05644.43 512-9 10.1097/01.ede.0000229155.05644.43 16804473

# Response

**Published:** 2022-03-02

**Authors:** Shatabdi Goon

**Affiliations:** University of Illinois at Urbana-Champaign, USA

The authors provided some PAR estimates for the dietary and lifestyle risk factors associated with CRC and BC in the UK along with its constituent countries. I couldn’t help wondering about the rationale underlying this study and several methodologic issues after reading this manuscript.

1. The rationale of carrying out this study eludes me, especially a large chunk of the result was just a repetition of Brown et al. (Br J Cancer, 2018, 118(8): 1130–41). The PAR for individual and combined risk factors of CRC and BC can be found in the supplementary materials of Brown’s paper, except for dietary calcium and red meat. Was the rationale of this study to update the PAR estimates in the UK by including these two risk factors, which were excluded in Brown’s paper?

**Author’s response:** The rationale was to conduct the PAR based on up-to-date criteria from the World Cancer Research Fund/American Institute of Cancer Research (WCRF/AICR).   This other analysis by Brown et al did not account for red meat or dietary calcium, now considered as probable risk factors by WCRF/AICR, and included radiation and oral contraceptive use, which we did not consider. While technically modifiable, radiation on OC use is not considered “lifestyle” in the same way as diet.  In addition, we showed results including and excluding smoking, which is an important risk factor but not in the WCRF/AICR purview of nutrition, physical activity, and body weight. While there are similarities with the Brown et al paper, our main goal was to calculate the PAR estimate for the most up-to-date data from the WCRF/AICR for of nutrition, physical activity, and body weight.

  

1. The authors obtained exposure distribution data on dietary calcium and red meat consumption from EPIC-Oxford cohort and UK biobank cohort (page 3). However, these two cohorts are not population representative of UK. For example, the EPIC-Oxford cohort clearly stated on their website that the sample is not representative of the general population, as 50% of their sample are vegetarians. ( http://www.epic-oxford.org/faq/1262/frequently-asked-questions). Similarly, UK Biobank participants were more likely to be older, to be female, and to live in less socioeconomically deprived areas than nonparticipants (Fry A. et al., Am J Epidemiol. 2017, 186(9):1026-34 ^2^ ). Therefore, the PAR estimates of calcium and red meat are bound to be biased using the data.

**Author’s response:  **We agree that both EPIC and UK biobank is not population-representative like UK survey data. Due to prevalence data unavailability on red/proceed meat and calcium intake- we planned to use other large UK-based cohorts (i.e., UK biobank and EPIC). Diet in both UK biobank and EPIC is somewhat healthier side on average; we have probably slightly underestimated PAR.

1. I wonder why the authors did not obtain cancer incidence data from the cancer registries in the UK. Without the incidence data, there are at least three limitations regarding the PAR estimates from this study. First, the authors could not estimate the attributable cancer cases, which is a constituent part of PAR estimation. Second, the authors could not do an age-stratified analysis. The summation of attributable cases of the age groups, divided by the cancer incidence of all age groups, provides a more accurate all-age PAR estimate than directly using an all-age prevalence to estimate PAR (the latter of which I suspect is what the authors did). This is because some risk factors are more prevalent among older age groups (e.g., physical inactivity, body fatness) and most cancer incidences are disproportionally higher among older age groups. Second, the authors had to assume a weighted average of 70% colon and 30% rectal cancers for physical activity and CRC (page 5), which wouldn’t be necessary if they had the incidence data by colon and rectum.

**Author’s response:** The relative risk estimates are based on the literature and not given in age stratification typically. The method relies on two parameters, the relative risk and the prevalence of the exposure. The proportion is what is calculated, not the absolute number of cancers estimated. Of course, the proportion can be multiplied by the incidence to get the absolute numbers. The 70-30 assumption was made based on the paper was based on the RR estimated for colon cancer is 0.84 and that for rectal cancer 0.87.  We assumed that for colorectal cancer, the RR would be a weighted average of these, which is around 0.85 (i.e., if the RR for colon cancer is 0.84 and that for rectal cancer around 0.87, the RR for CRC would be around 0.85). Because these values are so close anyway, this assumption appears reasonable. The 70-30 prevalence was assumed based on published data.  (Siegel RL, Miller KD, Goding Sauer A, Fedewa SA, Butterly LF, Anderson JC, Cercek A, Smith RA, Jemal A. Colorectal cancer statistics, 2020).

1. The authors didn’t correctly estimate the PAR of body fatness and physical activity for BC. These two risk factors are only associated with postmenopausal breast cancers, whereas the authors included premenopausal breast cancers as well.

**Author’s response**: Incidence data do not distinguish pre- and post-menopausal breast cancer, so these cannot be separated directly. Physical activity is considered a (protective) risk factor by the WCRF/AICR for both pre- and post-menopausal breast cancer. The only factor that is different is body fatness, which is considered a risk factor only for postmenopausal women. Assuming that body fatness is only a risk factor for postmenopausal breast cancer, they account for about 75% of cases. Since one of the four factors is not relevant for 25% of the cases, our estimate may be overestimated by around 5%.

  

1. The way the authors calculated RRs for calcium and physical activity is difficult to understand. They used a formula that assumes a loglinear dose-response relationship and calculates RR based on per unit RR, which is a usual practice in this field. Then for dietary calcium, they calculated an average RR for 0–199 mg/day, 200–399 mg/day, and 400–599 mg/day from their IJC paper (Kim H. et al., Int J Cancer, 2021, 148(12):2947-53 ^3^ ) to match 0–524 mg/day of the lowest category of exposure in this study (page 5, first paragraph). It was unclear to me how they averaged the RR and how they resolved the issue that the highest category (400-599mg/day) exceeds the upper limit (524 mg/day). Why not just take the midpoint value of this category (262 mg/day) and combine it with the risk per unit value? For example, for a reference calcium level of 1000 mg/day and per mg/day unit RR of r_mg, the RR for the category of 0-524 mg/day is r_mg^(1000-262).

**Author’s response:** Because of the exposure data unavailability that matches with the RR- we had to average RR to match with a specific category of intake. Exposure data wasn’t specified in the cohort we used data from the way we categorized to match with RR. 

 Some other concerns:

1. The authors used a formula, exp(lnX/A *B) to calculate RR, while neither A nor B was defined. The authors referenced their recent publication in IJC for this formula (Kim H. et al., Int J Cancer, 2021, 148(12):2947-53 ^3^ ). But in their IJC paper, the formula was laid out as “RR_B = exp(log(RR_B)/A *B)”, which is simply wrong. “log(RR_B)” should be “log(RR_A)”. The authors should write an erratum to IJC to have it corrected.

**Author’s response:** This was a typo in the PMC version. Now it is fixed.

  

1. In the second paragraph of the Method section, the author stated, “the prevalence of exposures to risk factors in the populations was obtained from the nationally representative population surveys” (page 3). However, which risk factors exactly were not stated. This caused some confusion to me, as I tried to figure out from which data source they obtained the prevalence of processed meat consumption.

**Author’s response:** Meat and calcium were not from the nationally representative survey, data sources were mentioned in the table as a footnote.

  

1. In Table 2, there is no variation in PAR by countries for red meat and dietary calcium (page 6). It is unclear whether there is indeed no country-level variation for these two risk factors, or there were no prevalence data to support this analysis, or there was a data-entry mistake. The authors didn’t have any discussions around it. If there were no prevalence data to support this analysis, then the discussion around differences in PAR between countries should acknowledge this limitation.

**Author’s response:** Prevalence data were only for the UK- not for country-specific.

1. At the end of the second paragraph of the introduction (page 3), the authors stated that other contributors to the variability in calculated PAR estimates by different studies differences include “socio-demographic profile of cancer cases and differences due to screening availability”. As the authors didn’t provide citations to support this statement, could the authors further elaborate on how these two factors contribute to the variability? In particular, it is difficult to fathom how screening availability, a factor related to the secondary intervention of CRC and BC, contributes to the PAR of diet and nutrition.

**Author’s response:** Screening is typically associated with the socio-demographic profile, and thus often with the dietary and lifestyle factors that we assessed. The broad effect of colonoscopy is to lower colorectal cancer risk (by removing precursors) and mammography tends to lead to more cases diagnosed (“overdiagnosis”). How these affect our results is complex; for example, they may affect the initial RR estimates (confounding).

  

1. The authors used an equation that has been widely adopted for estimating combined risk factors (page 5, paragraph 4). However, many researchers used this formula without disclaiming the two important assumptions associated with it (Steenland and Armstrong, 2006, Epidemiology, 17(5):512-9 ^4^ ): independent risk exposure distributions and no statistical interactions between any two risks. These two assumptions should not only be explicitly stated in the method section but also be worthy of several sentences in the discussion section. Also on page 5 after the formula, the author said “where i signifies the level of individual categories (i = 1,…, n).” “Categories” here should be “risk factors”. Again for this paragraph, the first sentence “We then estimated the preventability of CRC or BC that was attributable to the combined dietary and lifestyle risk factors the following equation” is missing a preposition.

**Author’s response:** We agree that these are important assumptions for the formula. It has been fixed in the revised one. 

  

1. Page 5 line 10, “600 METs-hours/week” should be “600 MET-m/week”. (fixed) 
2. In summary, I find little value being added by this paper beyond the study by Brown et al. (2018). The authors should justify the validity of their PAR estimates for dietary calcium and red meat and address all my concerns above before the paper could be considered having reasonable quality.
**Author’s response:** Because nationally representative exposure data were not available for meat and calcium – we had to use a large UK-based cohort (Biobank and EPIC). This may have caused a slight underestimate in the PAR estimate.
